# Serological prevalence and public health significance of brucellosis on a dairy farm in Namibia from 2011 to 2014

**DOI:** 10.1186/s13104-017-2933-x

**Published:** 2017-11-25

**Authors:** Oscar Madzingira, Precious Mupoti Sezuni

**Affiliations:** Department of Animal Health, University of Namibia, P. Bag 1096, Ngweze, Katima Mulilo Namibia

**Keywords:** Brucellosis, Dairy, Prevalence, Public health

## Abstract

**Objective:**

The main objective of this study was to determine the serological prevalence of brucellosis on a dairy farm with no past history of abortions, but where *Brucella* control measures including test and slaughter and vaccination of heifers at 4–8 months of age was practiced. Secondary data from 2011 to 2014 obtained from the Epidemiology Section of the Directorate of Veterinary Services was used.

**Results:**

Mandatory annual brucellosis testing results for mature dairy cows on a dairy farm for the period 2011–2014 were collated and analyzed. Results of a total of 6912 cows were analysed. The data comprised of the year of testing, number of cows tested for *Brucella* antibodies and the number of cows that tested positive. Serological testing was carried out using the Rose Bengal Test (RBT) as a screening test and the Complement Fixation Test as a confirmatory test for results that tested positive on the RBPT. Over the 4-year period, one dairy cow tested positive for *Brucella* antibodies in 2013 giving an apparent prevalence of 0.05% and an overall prevalence of 0.01%. When apparent prevalence was adjusted for RBPT test specificity and sensitivity of 71 and 78% respectively, true prevalence was determined to be zero.

## Introduction

Brucellosis is a zoonosis of major public health, animal welfare and economic significance [1]. It is endemic in most African countries [2]. A number of biovars of *Brucella abortus*, *B. melitensis* and *B. suis* are responsible for the disease in domestic animals [1] and zoonotic disease. *Brucella abortus* and *B. melitensis* are common causes of brucellosis in cattle, sheep and goats respectively [3]. Of the three *Brucella* species, *B. melitensis* causes the most severe disease in humans. In Namibia, *Brucella melitensis* was first reported in Karakul sheep [4].

Infection in humans is commonly acquired through direct and indirect contact with infected material such as aborted foetuses and the consumption of raw milk and unpasteurised dairy products including soft cheeses [5]. The disease in humans may result in abortions in pregnant women, infertility or a chronic debilitating disease. Available evidence suggests that persons infected with the Human Immunodeficiency Virus (HIV) are at a greater risk of severe brucellosis [6].

The economic significance of brucellosis results from production losses associated with abortions, mastitis, milk fever, retained placenta, metritis, impaired fertility and arthritis [1]. Milk production losses in infected dairy cows can be up to 20% and the inter-calving period can be prolonged by several months [2].

In Namibia, brucellosis has been reported in sheep [7]; sheep and springbok ([8–10] and other wild ruminants [11]. However, there is limited information on the prevalence of the disease in dairy cattle that are a source of raw milk to a greater part of the Namibian population. Therefore, this study was carried out to determine the serological prevalence of brucellosis on a dairy farm so as to make inferences about the potential exposure of consumers of raw milk to brucellosis and to provide a basis for future studies on bovine brucellosis.

## Main text

### Methods

#### Study farm and animals

The study farm was located south of the veterinary cordon fence and was one of the two major dairy enterprises in the country with about 2000 dairy cows and heifers. Milk produced from the farm was processed and sold as fresh or pasteurised. Sera for the brucellosis testing program were obtained from sexually mature Friesian–Holstein cows of various ages, at different stages of lactation and reproductive status fed on irrigated pastures. Heifers were not part of the testing program. The numbers of animals on the farm fluctuated each year due to introductions of replacement heifers or the exit of culled cows from the herd.

#### Data collection

Data for this retrospective study was obtained from the Epidemiology Section of the Directorate of Veterinary Services with permission of the dairy farm. The data from 2011 to 2014 comprising of the year of testing, number of cows tested for *Brucella* antibodies and the number of cows that tested positive was collated and analysed using simple descriptive statistics in Microsoft Excel^®^ program to estimate annual and overall brucellosis prevalence.

#### Testing of sera

Sera were screened for *Brucella* antibodies using the Rose Bengal Test (RBT). Sera testing positive on the RBT were confirmed using the Complement Fixation Test (CFT) following the procedure described in the OIE Manual [12]. In the RBT test, any agglutination observed was considered as test positive. In the CFT test, if haemolysis was present, the sample was considered negative, and the absence of haemolysis as indicated by a red button in the centre of the well, was considered positive as described by Chisi et al. [13].

## Results

Results of this study are summarised in Table 1. A total of 6912 cows were tested for *Brucella* antibodies over the 4-year period. One dairy cow tested positive for *Brucella* antibodies in 2013 with a titre of 1:8 giving an apparent prevalence of 0.05% and no positive reactors were recorded in 2011, 2012 and 2014. Overall prevalence was 0.01%. Apparent prevalence (annual and overall) were adjusted according to Reiczigel et al. [14] using RBPT test specificity and sensitivity of 71 and 78% [15] respectively and were found to be zero in both cases.

**Table 1:** Annual *Brucella* testing results and prevalence

Year	Number of sera tested	Number of sera positive	Prevalence (%)
2011	1657	0	0
2012	1681	0	0
2013	1872	1 (1:8)	0.05
2014	1702	0	0

### Discussions

A total of 6912 Friesian–Holstein cows were tested for brucellosis from 2011 to 2014. These were cows in the same herd that were repeatedly tested for brucellosis every year for 4 years. The number of dairy cows tested per year fluctuated with the number of replacement heifers introduced and cows culled from the herd. Over the 4 year period, one cow tested positive for *Brucella* antibodies in the year 2013 giving a low annual and overall apparent prevalence of 0.05 and 0.01% respectively. True prevalence calculated by adjusting apparent prevalence for RBT test sensitivity and specificity according to Reiczigel et al. [14] was determined to be zero indicating that the positive test may have been a false positive. Cross reactions with other organisms such *Yersinia enterocolitica* O:9 [16–18] and the vaccination of heifers between 4 and 8 months of age could have been responsible for the positive reactor. Heifers were vaccinated using *Brucella* S19 vaccine at 5 months according to the compulsory vaccination protocol. The S19 vaccine can result in the production of persistent antibodies which can cause false positive cases in serological tests [19]. Heifers were vaccinated using *Brucella* S19 vaccine at 5 months according to the compulsory vaccination protocol. It should be noted that no serological test gives an absolutely accurate result. Diagnosis should be based on two or more tests [20].

Other studies carried out on commercial dairy farms using RBT as a screening test and CFT or i-ELISA as confirmatory test, reported individual cow prevalence of 0.70–5.5% [20], 1.2% [21], 1.3% [22], 1.4% [23], 1.5% [24], 1.7% [25] and 1.9% [26]. The low prevalence recorded in our study is in agreement with low prevalence recorded on commercial dairy farms in other countries.

The positive reactor, an 8 year old cow was culled as part of the national protocol for controlling brucellosis on dairy farms which is based on the test and slaughter policy. After culling the positive cow in 2013, the herd was free of the disease in 2014. However, for as long as the cow was in the herd, the risk of humans contracting brucellosis through raw milk was present. The absence of reactors in 2011 and 2012, suggests that the infection in the cow may have been a recent one or a false positive. However, this could also be a reflection of the lower and variable sensitivity of the RBT (63–99%) which missed the infection in 2011 and 2012 or the weakness of the test in detecting brucellosis in recently aborted cows or chronically infected cows [27, 28]. It has been reported that *Brucella* antibodies fluctuate in circulation at different phases of infection [19] and this can make it difficult to detect infected cows in low prevalence herds using tests with low sensitivity and specificity. The RBT and CFT tests used for serological screening and confirmation in this study are recognised tests for international trade purposes [12, 29], but the RBT has a low specificity in herds with a low brucellosis prevalence [29] as in the present study.

According to Matope et al. [30] and Mai et al. [31], effective measures for brucellosis control in bovines include quarantine and testing of new arrivals, calf hood vaccination, culling of positive reactors, controlled grazing, use of screened semen for insemination and the implementation of biosecurity measures. Dairy cows in our study were managed intensively under strict biosecurity protocols as described by Matope et al. [30] and Mai et al. [31]. Therefore, the likelihood of introducing *Brucella* infections from outside the farm was low and this may explain the low serological prevalence recorded.

The low prevalence of brucellosis reported on the dairy farm confirms that the mandatory measures enforced by the state veterinary services to control the disease on the farm that are based on the test and slaughter approach were effective. The test-and-slaughter approach, as applied on the dairy farm, has been reported to be effective in herds with a low brucellosis prevalence [32, 33] as reported in our study. Based on the findings of this study, it can be concluded that the risk of animal handlers and consumers of raw milk contracting brucellosis from this farm is minimal, but cannot be excluded. It is therefore recommended that animal handlers be trained on the potential risk and that they put on the necessary personal protective clothing at all times when handling dairy cows. Consumers need to prioritise the boiling of raw milk before consumption or the consumption of pasteurised milk as a safe alternative because current serological tests are not absolute. The potential risk of zoonosis from dairy cows and fresh milk needs to be reflected in legislation and policy on occupational health and on trade in fresh milk in the country. The use of diagnostic tests with a higher specificity and sensitivity such as a Competitive ELISA (c-ELISA) is recommended in low prevalence herds is recommended. Results of this study are consistent with the fact that the farm had no clinical history of brucellosis as confirmed by records obtained from the regional state veterinary office.

The lack of information on age, parity and history of abortions precluded the assessment of risk factors for *Brucella* positivity. Due to the absence of records of replacement heifers, culled cows and the exact identity of animals tested for brucellosis annually, it was not possible to identify cows that were repeatedly tested over the study period.

## Limitations

- The study was carried out on one commercial dairy farm.
- The age and parity of individual cows was not available.
- There were no records of abortions.
- There were no records of the number of replacement heifers introduced and the number of cows culled and the reasons thereof.

## Abbreviations

RBT: Rose Bengal Test
CFT: Complement Fixation Test
HIV: Human Immunodeficiency Virus
OIE: World Organisation for Animal Health
FAO: Food and Agriculture Organisation
c-ELISA: Competitive Enzyme Linked Immunosorbent Assay
i-ELISA: Indirect Enzyme Linked Immunosorbent Assay
